# Use of the Maslach Burnout Inventory Among Public Health Care Professionals: Protocol for a Scoping Review

**DOI:** 10.2196/42338

**Published:** 2022-11-01

**Authors:** Juliana Pontes Soares, Rayssa Horacio Lopes, Paula Beatriz de Souza Mendonça, Cícera Renata Diniz Vieira Silva, Cláudia Cristiane Filgueira Martins Rodrigues, Janete Lima de Castro

**Affiliations:** 1 Postgraduate Program in Collective Health Federal University of Rio Grande do Norte Natal Brazil; 2 Postgraduate Program in Collective Health Federal University of Espírito Santo Vitória Brazil; 3 Technical School of Health of Cajazeiras Federal University of Campina Grande Cajazeiras Brazil; 4 School of Health Federal University of Rio Grande do Norte Natal Brazil

**Keywords:** health care personnel, health care workers, public health services, Maslach burnout inventory, burnout, health care professional, workplace stress, mental health, occupational health, psychological well-being, policymaker

## Abstract

**Background:**

Burnout syndrome is a chronic response to stressors in the workplace. It is characterized by emotional exhaustion and physical and mental burnout and may lead to high employee turnover, work absenteeism, and increased occupational accidents. Most studies use the Maslach Burnout Inventory (MBI) to identify burnout and implement preventive actions and treatments.

**Objective:**

This study presents a scoping review protocol to identify and map studies that used MBI to assess burnout syndrome in health care professionals working in public health services.

**Methods:**

This scoping review protocol follows the Joanna Briggs Institute reviewers’ manual, and this protocol consists of 6 stages: identifying the research question, identifying relevant studies, study selection, data extraction and coding, analysis and interpretation of results, and consultation with stakeholders. We will conduct searches in Embase, LILACS, PubMed/MEDLINE, PsycINFO, Scopus, Web of Science databases, and gray literature. The main research question is as follows: how is MBI used to identify burnout syndrome in health care professionals working in public health services? Inclusion criteria will comprise qualitative and quantitative studies using MBI to identify burnout syndrome in health care professionals working in public health services and no restrictions in language and publication dates. Data will be extracted using a spreadsheet adapted from the Joanna Briggs Institute model. Quantitative and qualitative data will be analyzed using descriptive statistics and thematic analysis, respectively. The consultation with stakeholders will be essential for increasing the knowledge about MBI, identifying new evidence, and developing future strategies to guide public policies preventing burnout syndrome in health care professionals working in public services.

**Results:**

This protocol will guide a scoping review to identify and map studies that used MBI to identify burnout syndrome in health care professionals working in public health services. The results of this review may be useful to public health care professionals, managers, policymakers, and the general population because these findings will help understand the validated, translated, and adapted versions of MBI and domains, number of items, Likert scales, and cutoff points or the latent profile analysis most used in the literature. Furthermore, possible research gaps may be identified to guide future studies. All information regarding the stages of the scoping review favor its transparency and allow it to be methodologically replicated according to the principles of open science, thereby reducing the risk of bias and data duplication.

**Conclusions:**

This study may reveal the multiplicity of scales described in the literature and the different forms of assessing burnout syndrome in health care professionals. This study may help to standardize the assessment of burnout syndrome in health care professionals working in public health services and contribute to the discussion and knowledge dissemination about burnout syndrome and mental health in this population.

**International Registered Report Identifier (IRRID):**

DERR1-10.2196/42338

## Introduction

Burnout syndrome is a work-related psychological syndrome included in the International Classification of Diseases 11th Revision (code QD85) [1]. Herbert Freudenberger described this syndrome in 1974 [2,3], and it is characterized by a chronic response to interpersonal stressors in the workplace that may be related to work organization and environment [4,5]. The protocol in our study will guide a scoping review to identify and map studies that used the Maslach Burnout Inventory (MBI) to identify burnout syndrome in health care professionals working in public health services. The results of this review may be useful to public health care professionals, managers, policymakers, and the general population since these findings will help understand the validated, translated, and adapted versions of MBI and domains, number of items, Likert scales, and cutoff points or the latent profile analysis most used in the literature. Furthermore, possible research gaps may be identified to guide future studies. All information regarding the stages of the scoping review favor its transparency and allow it to be methodologically replicated according to the principles of open science, thereby reducing the risk of bias and data duplication [3].

Different definitions of the burnout syndrome consider many etiological factors; thus, literature lacks a consensus about its definition and diagnostic criteria [6,7]. Some authors define burnout syndrome as fatigue and emotional exhaustion [8], whereas others consider it as emotional exhaustion and depersonalization [9]. Gil-Monte [10] defines burnout syndrome according to 4 dimensions: enthusiasm toward the job, psychological exhaustion, indolence, and guilt. Bakker et al [11] define the syndrome as mental distance from the job and emotional, physical, and cognitive exhaustion. One of the most used definitions of burnout syndrome was proposed by Maslach and Jackson who considered this syndrome as emotional exhaustion, depersonalization, and personal accomplishment associated with physical and psychological symptoms. Thus, this scoping review protocol used this definition to map the use of MBI [5].

The main characteristics of burnout syndrome are emotional exhaustion, resulting in fatigue; depersonalization, associated with negative behaviors and cynicism in work relationships; and low scores in personal accomplishment, with feelings of incompetence and low productivity [12-14]. Individuals with burnout syndrome see work as a source of misery and unpleasurable [3], leading to high employee turnover, work absenteeism, decreased quantity and quality of work, and increased occupational accidents, thereby representing institutional consequences [15,16]. People working with the general public are more prone to develop the burnout syndrome. Studies have shown burnout in several work fields; however, the most affected were teachers, police officers, and health care professionals (especially physicians and nurses) [6,17-19]. The burnout syndrome affects approximately 10%-70% of nurses and 30%-50% of physicians, nurse practitioners, and physician assistants [20] and is highly prevalent among health care professionals [21-24], especially among those working in public health services [25,26].

Many aspects of burnout syndrome have already been established, and more than 90% of the studies used MBI [27] to identify and implement preventive interventions and treatments [12,28]. MBI is a self-report questionnaire developed by Christina Maslach and Susan Jackson to obtain a multidimensional view of burnout and comprises 22 items divided into 3 sections: emotional exhaustion (9 items), depersonalization (5 items), and personal accomplishment (8 items). The original version uses a 7-point Likert scale (0 corresponds to “never” and 6 to “every day”) [5,29] that assesses burnout according to scores in each dimension: high levels of burnout are characterized by high scores in emotional exhaustion and depersonalization and low scores in personal accomplishment. According to studies, MBI must be used in a 3D approach to enhance the comprehension of the burnout syndrome, since all 3 dimensions are considered important for identifying burnout and the individual dimensions alone do not sufficiently define the construct [21,30-32].

MBI is also intended to investigate the syndrome in other work settings. The MBI-Educators Survey is intended for education professionals, and the MBI-General Survey measures the syndrome in any professional category. For health care professionals, there is a specific version called the MBI Human Services Survey [5,29]. The MBI-Student Survey is designed to identify burnout in students [33]. The literature shows different translated, validated, and adapted versions of MBI. This diversity constitutes a challenge for the development of research using the instrument, since it can impact the interpretation of data and make comparisons between studies impossible, as well as hinder the identification of workers at risk for developing the syndrome because there is no standardization in its use.

The choice of the setting in our study was due to evidence in the literature that professionals working in public health services are more exposed to stress, thereby causing burnout [34]. In these services, there is usually wage-related dissatisfaction on the part of professionals, the need for better planning of actions and work processes [35,36], the difficulty in operationalizing actions, underfunding, and poor infrastructure [35-37]. The evidence of the aforementioned factors in public health services should be considered and will help us in terms of understanding that the development of burnout syndrome in health care professionals may be related to the organization and development of the work process in these services, which reflects the importance of conducting this study in the current context. Considering the global importance of MBI for identifying burnout syndrome [38], we conducted a preliminary search in the Joanna Briggs Institute (JBI) evidence synthesis, the international Prospective Register of Systematic Reviews (PROSPERO), Cochrane Library, and PubMed/MEDLINE databases by using the descriptors “health care professionals,” “public health services,” “Maslach Burnout Inventory,” and “burnout syndrome.” We found only 1 integrative review synthesizing the use of MBI in Brazil; however, this study was not directed to public health care professionals [39]. No studies or protocols were found when expanding the search to other continents.

Therefore, this scoping review protocol will identify and map studies that used MBI to assess burnout syndrome in health care professionals working in public health services. It will allow us to map the evidence describing different translated and validated versions of MBI and assess the consistency in frequencies and cutoff points of the scale and its application in different countries, aiming to guide a possible standardization in its use. We will also evaluate possible research gaps regarding the use of MBI among health care professionals working in public health services.

## Methods

### Overview

**Figure 1:**
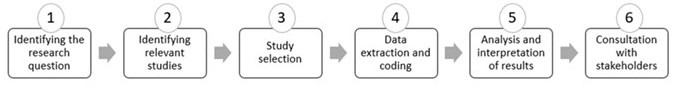
Stages of the scoping review.

This scoping review protocol follows the JBI reviewers’ manual [40] and is guided by the PRISMA-ScR (Preferred Reporting Items for Systematic Reviews and Meta-Analyses Extension for Scoping Reviews) [41]. This protocol is registered in the Open Science Framework [42]. Our review will follow 6 stages [43,44], as shown in Figure 1.

### First Stage: Identifying the Research Question

The research question was formulated using the Population, Concept, Context mnemonic [40] (Table 1). The Population, Concept, Context mnemonic maps information to identify knowledge gaps, present key concepts, quantify aspects of interest, and identify practices and evidence in the thematic area [45].

In this context, the questions of this scoping review protocol were defined as follows: “How is MBI used to identify burnout syndrome in health care professionals working in public health services?”, “What is the most studied professional category using MBI?”, “What are the main results of MBI in health care professionals working in public health service?”, and “What are the recommendations for clinical practice arising from the use of MBI?”

**Table 1:** Definition of concepts used in the review.

Research question theme		Definition
**Population: health care professionals in public services**		
	Health care professionals	Workers of health care services, with or without professional qualification, working or not in health programs and institutions, and subjected or not to government regulation
	Public health services	Responsibilities of the state government regarding health care. It includes responsibilities for executing activities and specific actions in public health, or mobilization, promotion, orientation, and articulation of social agents [46]
**Concept:** **use of the Maslach Burnout Inventory**		
	Maslach Burnout Inventory	Instrument used to investigate burnout syndrome in workers [29]
**Context: health care professionals with burnout syndrome**		
	Burnout syndrome	Psychological syndrome characterized by emotional exhaustion, depersonalization, and reduced personal accomplishment and present in individuals working with the public [29]

### Second Stage: Identifying Relevant Studies

The search strategy was based on 4 controlled vocabularies in health: health sciences descriptors, medical subject headings, Emtree, and the American Psychological Association thesaurus. Natural language processing was used to increase sensitivity and expand the search results [47,48]. A librarian also refined the search strategy to increase the number of results in all the databases. The strategy was built using a high sensitivity model composed of extraction, conversion, combination, construction, and use [47]. Table 2 shows the conversion of the mnemonics into descriptors.

**Table 2:** Conversion of mnemonics.

Mnemonic	Extraction	Conversion
Population	Health care professionals working in public health services	Health care personnel Public health services
Concept	Use of the Maslach Burnout Inventory	Maslach Burnout Inventory
Context	Health care professionals with burnout syndrome	Burnout

A description of the complete search strategy for PubMed/MEDLINE is shown in Multimedia Appendix 1. An initial exploratory search was performed in PubMed/MEDLINE to identify the main medical subject headings related to the topic. The search strategy combined the Boolean operators AND and OR and will be further adjusted to each database (Embase, LILACS, PubMed/MEDLINE, PsycINFO, Scopus, and Web of Science). The grey literature will be explored in ProQuest Dissertations & Theses Global, Google Scholar, Brazilian Digital Library of Theses and Dissertations, and Open Access Theses and Dissertations. Additional sources will also be manually retrieved from reference lists. If needed, authors will be contacted for additional information.

### Third Stage: Study Selection

The following inclusion criteria will be adopted: quantitative and qualitative full-text studies using MBI among health care professionals working in public health services and no language restriction and no publication date restriction. Duplicated studies, literature reviews, letters, editorials, theoretical essays, opinion articles, and studies analyzing burnout syndrome in non–health care professionals or private services will be excluded. Relevant studies will be retrieved and exported to a spreadsheet. Duplicated studies will be manually excluded. Two independent researchers will read titles and abstracts, and a third reviewer will be consulted in case of disagreement. Texts will be analyzed according to inclusion criteria, and reasons for exclusion will be registered and reported. Following the JBI manual, a pilot test with a random sample of 25 studies (title and abstract) will be conducted to assess eligibility criteria and agreement among the researchers enrolled (it must reach at least 75% of agreement before proceeding with independent assessment) [40]. Details regarding study selection (identification, screening, eligibility, and inclusion) will be presented in a flowchart [41].

### Fourth Stage: Data Extraction and Coding

Two independent reviewers will chart data by using an adapted data extraction form based on the JBI model [35] (Table 3).

**Table 3:** Instrument for data extraction.^a^

Variable	Standardization
Study type	Article, dissertation, or thesis
Publication date	Year of publication
Publication context	Where the study was conducted/published
Journal	In which journal the study was published
Author qualification	Major degree of the first author
Aim/purpose	Aim or purpose of the study
Research type	Research type described by authors
Data collection	Methods for data collection
Study population	Health care professionals and sample size
Sample	Number of participants
Maslach Burnout Inventory	Version of the Maslach Burnout Inventory used
Maslach Burnout Inventory domains used	Emotional exhaustion, depersonalization, and personal accomplishment
Items and Likert scale	Number of items and Likert score
Cutoff points or Latent profile analysis	Cutoff points to identify burnout syndrome or Alternative methods for identifying burnout
Results	Main results of the study
Challenges and limitations	Description of challenges and limitations in using the scale
Recommendations for practice Administration method	How the study contributes to improving the mental health of health care professionals In-person or using digital tools

^a^Source: Adapted from Joanna Briggs Institute model [41].

### Fifth Stage: Analysis and Interpretation of Results

Quantitative data will be analyzed using descriptive statistics and presented in absolute or relative frequency, whereas qualitative analysis will identify meanings and patterns by using thematic analysis [49].

### Sixth Stage: Consultation With Stakeholders

Preliminary results will be presented to 5 researchers in the field of burnout syndrome to contribute to knowledge dissemination, disclosure of findings, and identification of gaps in the use of MBI, frequencies, and cutoff points adopted worldwide. This step will be essential to increase knowledge about MBI, identify new evidence, and develop future strategies to guide public policies preventing burnout syndrome in health care professionals from public services [45]. An email containing the written informed consent and an electronic form with preliminary results will be sent to stakeholders. Interested parties will not be identified, and authors will ask for an analysis of the results.

### Ethics Approval

This study followed the ethical principles in human research and was approved by the research ethics committee of the Onofre Lopes University Hospital (4.952.319 and CAAE 46284921.4.0000.5292) on September 3, 2021.

## Results

This protocol will guide a scoping review to identify and map studies that used MBI to identify burnout syndrome in health care professionals working in public health services. The results of this review may be useful to public health care professionals, managers, policymakers, and the general population since the findings will help understand the validated, translated, and adapted versions of MBI and domains, number of items, Likert scales, and cutoff points or the latent profile analysis most used in the literature. Furthermore, possible research gaps may be identified to guide future studies. All information regarding the stages of the scoping review favor its transparency and allow it to be methodologically replicated according to the principles of open science, thereby reducing the risk of bias and data duplication.

## Discussion

### Principal Findings

This protocol will guide a scoping review to identify and map studies that used MBI to identify burnout syndrome in health care professionals working in public health services. This is one of the most important instruments used to identify burnout syndrome and is used worldwide, especially among health care professionals. In addition to the classification by cutoff points, this study advances the consideration of alternative methods for identifying burnout (latent profile analysis) to highlight significant associations and guide the identification of the number of profiles for a given construct. Through this analysis, it will be possible to explore whether the identified profiles differ from the clustering of MBI scores [50,51]. The protocol was developed by a research team with knowledge and experience in scoping reviews and by applying MBI to identify the burnout syndrome. A librarian helped develop a high-sensitivity search strategy based on the combination of 4 vocabularies and no date or language limitations to expand the results and allow a broad access to the literature. All information regarding the stages of the scoping review favor its transparency and allow it to be methodologically replicated according to the principles of open science, thereby reducing the risk of bias and data duplication.

### Limitations

As a limitation of the study, we emphasize that the multiplicity of MBI scales that can be found may reveal possible biases regarding the correct use of the instrument. In this sense, we will summarize the results according to the version of MBI most applied in each country and the populations studied. Although this protocol will guide the searches and development of the scoping review, it may not cover the entire literature; thus, studies indexed in different databases may not be found. Although we did not impose a language limitation for inclusion of the studies, the use of descriptors and search terms in English and Portuguese languages may be a limitation for this study.

### Conclusions

This study protocol presents the main methodological steps that will guide the scoping review and identify and map studies that used the MBI scale to identify the burnout syndrome in health care professionals working in public health services. This study may reveal the multiplicity of scales described in the literature and the different forms of assessing burnout syndrome in health care professionals. The results of our study may also help to standardize the assessment of burnout syndrome in health care professionals working in public health services and promote knowledge dissemination about burnout syndrome and mental health in this population.

## Appendix

Multimedia Appendix 1 Complete strategy for the search in MEDLINE/PubMed.

## Abbreviations

JBI: Joanna Briggs Institute
MBI: Maslach Burnout Inventory
PRISMA-ScR: Preferred Reporting Items for Systematic Reviews and Meta-Analyses-Extension for Scoping Reviews
PROSPERO: International Prospective Register of Systematic Reviews
