# Supplementary material for: Use of the Maslach Burnout Inventory Among Public Health Care Professionals: Protocol for a Scoping Review
Source: JMIR Res Protoc. 2022 Nov 1;11(11):e42338. doi: 10.2196/42338 (PMC9667379; doi:10.2196/42338)
Supplement: Multimedia Appendix 1 [file resprot_v11i11e42338_app1.docx]

**Multimedia Appendix 1. Complete strategy for the search in Medline/Pubmed.**

| **Search** | **Terms** | **Retrieved studies**  (Sept 8^th^, 2021) 15h41min |
| --- | --- | --- |
| **#1** | "health personnel" AND ("public health" OR "public health practice") AND ("burnout, professional" OR "burnout, psychological") [MeSH Terms] | 246 |
| #2 | "health care personnel"[Text Word] OR "health personnel"[Text Word] OR "health care practitioner"[Text Word] OR "health care professional"[Text Word] OR "health care professionals"[Text Word] OR "health care provider"[Text Word] OR "health care providers"[Text Word] OR "health care worker"[Text Word] OR "health care workers"[Text Word] OR "health profession personnel"[Text Word] OR "health worker"[Text Word] OR "health workers"[Text Word] OR "healthcare personnel"[Text Word] OR "healthcare practitioner"[Text Word] OR "healthcare professional"[Text Word] OR "healthcare provider"[Text Word] OR "healthcare providers"[Text Word] OR "healthcare worker"[Text Word] OR "healthcare workers"[Text Word] OR "public health officer"[Text Word] OR "allied health personnel"[Text Word] OR "paramedical personnel"[Text Word] OR "health care assistant"[Text Word] OR "health care support worker"[Text Word] OR "health support worker"[Text Word] OR "healthcare assistant"[Text Word] OR "healthcare support worker"[Text Word] OR "ophthalmic assistants"[Text Word] OR "para medical personnel"[Text Word] OR "paramedical assistant"[Text Word] OR "paramedical manpower"[Text Word] OR "paramedical professional"[Text Word] OR "paramedical staff"[Text Word] OR "psychiatric aides"[Text Word] OR "anatomists"[Text Word] OR "anatomist"[Text Word] OR anesthetists OR anesthetist OR anaesthesist OR "anaesthetic personnel"[Text Word] OR "anaesthetic staff"[Text Word] OR "anaesthetist"[Text Word] OR "anesthetic personnel"[Text Word] OR "anesthetic staff"[Text Word] OR "anesthetist"[Text Word] OR "audiologists"[Text Word] OR "audiologist"[Text Word] OR "caregivers"[Text Word] OR "caregiver"[Text Word] OR "care giver"[Text Word] OR "caregivers"[Text Word] OR "carer"[Text Word] OR "carers"[Text Word] OR "case managers"[Text Word] OR "case manager"[Text Word] OR "coroner"[Text Word] OR "coroners"[Text Word] OR "medical examiners"[Text Word] OR "procurator fiscal"[Text Word] OR "dental staff"[Text Word] OR "dentists"[Text Word] OR "dentists"[Text Word] OR "doulas"[Text Word] OR "doula"[Text Word] OR "labor coaches"[Text Word] OR "labor coach"[Text Word] OR "epidemiologists"[Text Word] OR "epidemiologist"[Text Word] OR "health facility administrators"[Text Word] OR "health facility administrator"[Text Word] OR "hospital personnel"[Text Word] OR "hospital employee"[Text Word] OR "hospital staff"[Text Word] OR "hospital staffing"[Text Word] OR "hospital worker"[Text Word] OR "infection control practitioners"[Text Word] OR "infection control practitioner"[Text Word] OR "infection control nurse"[Text Word] OR "medical chaperones"[Text Word] OR "medical chaperone"[Text Word] OR "patient chaperones"[Text Word] OR "patient chaperone"[Text Word] OR "medical laboratory personnel"[Text Word] OR "clinical laboratory personnel"[Text Word] OR "clinical lab personnel"[Text Word] OR "clinical lab technician"[Text Word] OR "clinical laboratory professional"[Text Word] OR "clinical laboratory scientist"[Text Word] OR "clinical laboratory technician"[Text Word] OR "medical lab personnel"[Text Word] OR "medical lab technician"[Text Word] OR "medical laboratory assistant"[Text Word] OR "medical laboratory personnel"[Text Word] OR "medical laboratory professional"[Text Word] OR "medical laboratory scientist"[Text Word] OR "medical laboratory technician"[Text Word] OR "medical personnel"[Text Word] OR "medical staff"[Text Word] OR "medical staffs"[Text Word] OR "hospitalists"[Text Word] OR "nurses"[Text Word] OR "nurse"[Text Word] OR "anaesthesist nurse assistant"[Text Word] OR "anesthetist nurse assistant"[Text Word] OR "community health nurse"[Text Word] OR "community health nurses"[Text Word] OR "nursing assistance"[Text Word] OR "public health nurse"[Text Word] OR "public health nurses"[Text Word] OR "nursing staff"[Text Word] OR "nursing staffs"[Text Word] OR "nurse staffing"[Text Word] OR "nursing manpower"[Text Word] OR "nursing personnel"[Text Word] OR "nutritionists"[Text Word] OR "dietitian"[Text Word] OR "dietician"[Text Word] OR "dieticians"[Text Word] OR "dietitians"[Text Word] OR "nutritionist"[Text Word] OR "nutritionists"[Text Word] OR "occupational therapists"[Text Word] OR "occupational therapist"[Text Word] OR "optometrists"[Text Word] OR "optometrist"[Text Word] OR "optometric technician"[Text Word] OR "hospital personnel"[Text Word] OR "hospital employee"[Text Word] OR "hospital staff"[Text Word] OR "hospital staffing"[Text Word] OR "hospital worker"[Text Word] OR "pharmacists"[Text Word] OR "pharmacist"[Text Word] OR "physical therapists"[Text Word] OR "physiotherapist"[Text Word] OR "physical therapist"[Text Word] OR "physiotherapists"[Text Word] OR "physician executives"[Text Word] OR "medical director"[Text Word] OR "medical directors"[Text Word] OR "physician executive"[Text Word] OR "physician"[Text Word] OR "physicians"[Text Word] OR "doctor"[Text Word] OR "medical doctor"[Text Word] OR "medical practitioner"[Text Word] OR "practitioner"[Text Word] OR "veterinarians"[Text Word] OR "veterinarian"[Text Word] OR "animal doctor"[Text Word] OR "veterinary physician"[Text Word]) AND ("public health services"[Text Word] OR "public health"[Text Word] OR "community health"[Text Word] OR "community health program"[Text Word] OR "community health programme"[Text Word] OR "national health"[Text Word] OR "national health programmes"[Text Word] OR "national health programs"[Text Word] OR "national health project"[Text Word] OR "public health system"[Text Word] OR "public health practice"[Text Word] OR "public health practices"[Text Word] OR "health care delivery"[Text Word] OR "delivery of health care"[Text Word] OR "delivery of healthcare"[Text Word] OR "health care supply"[Text Word] OR "healthcare delivery"[Text Word] OR "healthcare supply"[Text Word] OR "health care system"[Text Word] OR "health care system"[Text Word] | 659,589 |
| #3 | "maslach burnout inventory"[Text Word] OR "maslach burn-out inventory"[Text Word] OR "maslach burnout index"[Text Word] OR "maslach burnout inventories"[Text Word] OR "maslach burnout questionnaire"[Text Word] OR "maslach burnout scale"[Text Word] OR "maslach burnout scores"[Text Word] OR "maslachs burnout inventory"[Text Word] | 2,593 |
| #4 | "burnout"[Text Word] OR "burn-out"[Text Word] OR "burn-out syndrome"[Text Word] OR "burnout syndrome"[Text Word] OR "psychological burn-out"[Text Word] OR "psychological burnout"[Text Word] OR "professional burnout"[Text Word] OR "career burn-out"[Text Word] OR "career burnout"[Text Word] OR "occupational burn-out"[Text Word] OR "occupational burnout"[Text Word] OR "professional burn-out"[Text Word] OR "occupational stress"[Text Word] OR "job stress"[Text Word] OR "work stress"[Text Word] | 27,401 |
|  | # 1 OR #2 AND #3 AND #4 | 110 |
